# Age-related decrease in functional mobility score when performing a locomotor task in an immersive environment

**DOI:** 10.3389/fbioe.2023.1141507

**Published:** 2023-06-06

**Authors:** Alexandre Renaux, Fabien Clanché, Frédéric Muhla, Karine Duclos, Philippe Meyer, Sophie Colnat-Coulbois, Gérome Gauchard

**Affiliations:** ^1^ Development, Adaptation, and Handicap, Faculty of Medicine, Université de Lorraine, Lorraine, France; ^2^ CARE Grand Est, Research and Expertise Support Center, Nancy, France; ^3^ UFR STAPS, Faculty of Sport Science, Université de Lorraine, Lorraine, France; ^4^ Centre de Rééducation FLORENTIN, Nancy, France; ^5^ Centre de Recherche en Automatique de Nancy CRAN, Université de Lorraine, Campus Sciences, Lorraine, France

**Keywords:** immersive virtual reality, functional mobility, motor control, age dependence, locomotor tasks

## Abstract

In recent years, immersive virtual reality technology has emerged in the field of health. Its use could allow the assessment of the motor behavior of individuals in adaptable and reproducible immersive environments, simulating real situations. This study aimed to assess the effect of an immersive scenario on functional mobility during a simple locomotor task according to age. Sixty young adults and 60 older volunteers, who were autonomous and without cognitive and neurological impairment participated. A locomotor task based on the “Timed Up and Go” task was performed in real and virtual conditions. A functional mobility score was calculated by combining the time and the number of steps used and compared between young and older people. Results showed that correlations between time and the number of steps were the same in VR and real conditions, but the locomotor performance decreased significantly in VR for both populations. Additionally, older people exhibited a more reduced locomotor performance in a virtual environment than young adults, thereby their functional mobility score decreased more to complete the task, reflecting the adoption of a more secure locomotion strategy often related to the fear of falling, with an increase in time and number of steps to support balance. The major difference between reality and VR is the visual immersion with an HMD, and visual information is more important in the sensory integration of older people. Therefore, the reduction in visual field and lack of visual exproprioceptive information about the body segments in the virtual environment could explain these results. Finally, the effect of immersion in a virtual scenario on mobility exists for both populations but is accentuated by the aging process and is therefore age dependent.

## Introduction

The notable technological and technical development of virtual reality devices has allowed researchers and health professionals to successfully develop assessments and clinical treatments based on the use of these devices (Massetti et al*.*, 2018). Immersive virtual reality (IVR) is mainly based on the use of a head-mounted display (HMD) that allows the user to be engaged and immersed in a digitally created 360-degree virtual environment that can simulate several everyday situations. The user can perceive, explore, and interact in a pseudo-natural way (Fuchs, 2018), a numerical approach that simulates a real one, despite technical limitations, such as isolation from the real world, the limited field of view, the weight of the HMD, or the still improvable graphic quality (Renaux et al*.*, 2022). It engages people in sensorimotor and cognitive activities in a safe, reproducible, adapted, and controlled environment according to the objectives pursued (Abou et al*.*, 2020), and the motor behaviors can be measured. Virtual reality is favorably accepted by individuals who are inclined to use it for the benefits provided (Tuena et al*.*, 2020). For example, many studies have shown that patients in rehabilitation are more involved in programs using immersive virtual reality than in traditional programs (Kern et al., 2019) because they feel more competent, more autonomous, and more motivated, with less of a feeling of fatigue and high physical effort. These various advantageous aspects indicate that the possibilities of using immersive virtual reality in the field of health must be deepened and exploited.

Immersive virtual reality could thus be used as a new method to visually immerse an individual in a contextualized, standardized, and controlled virtual environment that reproduces a daily situation, and in which functional mobility capacities can be assessed. The evaluation of functional mobility is frequently performed by health professionals to determine the capabilities of individuals at a given time (Peel et al*.*, 2005). Functional mobility is a method for assessing individuals as it can provide information about their overall health status. Functional mobility is defined as the physiological ability of individuals to move independently and safely in a variety of environments (Forhan and Gill, 2013) and perform daily living activities or functional tasks, such as standing, bending, walking, or reaching for an object, which are essential for independent living and determining an individual’s overall health status. Reduced functional mobility is associated with an increased risk of falls, loss of independence, and institutionalization (Studenski et al*.*, 1994).

Different tests exist to assess functional mobility, such as the 6-m walk, coordinated stability, sit-to-stand (Butler et al*.*, 2009), and more specifically, the “Timed Up and Go” test (Podsiadlo and Richardson, 1991), which involves reproducing basic everyday actions, in this case getting up from a chair, walking 3 m back and forth, and sitting down. It is a highly recognized gold standard test used by health professionals and is considered as a quick and easy tool for assessing mobility, gait, balance, and fall risk (Alexandre et al., 2012). However, the main task requested is performed in an environment devoid of stimulation, which cannot reproduce the diversity of situations encountered in everyday life. IVR could thus be used to overcome this limitation, by allowing these actions to be performed in a contextualized, standardized, and enriched visual environment, which is more representative of daily life and could increase the level of sensibility and specificity.

To use IVR as part of a new assessment tool and interpret the data correctly, it is necessary to know the effects on motor behavior beforehand and to take them into consideration. Many studies have already been conducted and showed positively that visuomotor behavior is essentially similar between real and virtual environments; therefore, the participant uses known sensory motor patterns to evolve in the virtual environment. This allows motor behavior to be studied, evaluated, or trained in immersive situations (Fink, Foo and Warren, 2007; Gérin-Lajoie et al*.*, 2008; Bühler and Lamontagne, 2019). Nevertheless, an immersive virtual reality effect on the motor strategies used does exist and is linked to the technical constraints of the virtual reality device. This effect is measurable between the execution of the same task in reality and in virtual reality, especially on the parameters of static or dynamic balance (Robert, Ballaz and Lemay, 2016; D’Antonio et al*.*, 2020) but also on gait parameters, with an increase in the number of steps, stride speed variability, and step width (Hollman et al*.*, 2006), causing a decrease in walking speed (Fink, Foo and Warren, 2007; Agethen et al*.*, 2018). These motor adaptations in virtual reality, particularly during movement, have been measured in a population of young adults, whose abilities were optimal (Bovim et al*.*, 2020; Renaux et al*.*, 2022), and also in a population of older people, who had declining functional abilities (Muhla et al*.*, 2022). Therefore, there is an immersive virtual reality effect on motor parameters for all ages during a locomotor task, linked to the characteristics of the virtual experience.

Advanced age has a strong impact on motor skills because there is a progressive deterioration of motor, sensory, and cognitive functions, affecting posture, balance, mobility, and motor control compared with young adults (Blain et al*.*, 2015). The causes of these motor deficits are multifactorial and include the degradation of the central nervous system, modification of the sensory receptors necessary to decode the information of the environment, reduction of mass and muscular force, and degeneration of the peripheral nerves; all contribute to the aging process of (Seidler et al*.*, 2010). Additionally, it has been noted that older people exhibit a decrease in the quality of their motor coordination and an increase in the variability and slowness of the movements performed, unlike young adults. (Contreras-Vidal, Teulings and Stelmach, 1998; Raz et al*.*, 2003; Callisaya et al*.*, 2013). Furthermore, older people use sensory input differently and rely more on visual information for sensory integration (Agathos et al*.*, 2017). A fundamental difference between young adults and older people also lies in the psychological aspect, related to the decline in functional and cognitive abilities. Older adults can be strongly affected by the fear of falling on a daily basis, which can lead to a limitation of motor activity and a decrease in quality of life (Li et al*.*, 2003). The discovery of a new technology and a new environment, which is virtual, could lead to a more pronounced apprehension for them. Nevertheless, studies have shown a strong acceptance of virtual reality among the older people who find the device useful and easy to use (Delbes et al., 2022), with little difference from younger people (Ito et al*.*, 2019).

Previous studies have shown an effect of immersive virtual reality on motor behavior, with a degradation of locomotor performance in both older people and young adults. However, the magnitude of this decrease as a function of age has not been evaluated. In this context, the aim of this study is to quantify the age-related effect of immersive virtual reality on the decrease in locomotor performance. Cognitive and motor changes related to the aging process could intuitively have a greater influence on motor behavior in a virtual environment, and we hypothesized that there is a greater decrease in locomotor performance in older people compared with young adults.

## Materials and methods

### Participants

Sixty young adults aged from 18 to 25 were recruited in the Université de Lorraine, and sixty older people aged from 65 to 85 were recruited in the OHS Florentin rehabilitation center. Participants were autonomous, without cognitive or neurological impairment, and independent in walking with or without assistance. They all experienced IVR exposure for the first time. They volunteered to participate in the experiment. This study was approved by an ethical committee: CPP EST-III, N°ID-RCB: 2018-A02637-48. Participants gave their oral consent to participate.

### Experimental protocol

All participants were asked to perform a locomotor task consisting of a go back and forth of 3 m, starting and ending in a seated position, walking as quickly as possible with safety. The oral instruction given to the participants was “you must perform the task by walking as quickly as possible without running or putting yourself in danger”. When participants heard the word “go” they stood up, walked 3 m, turned around and returned to their initial position. It was a simple locomotor task inspired by the “Timed Up and Go” motor task by Podsiadlo and Richardson (1991). This task had the advantage of being relevant, recognized for assessing functional mobility, and simple to perform in reality and in virtual reality in a limited space of 4 m by 4 m with outside-in tracking. It was easily contextualized in a fully reproducible visual environment. The task was timed: the start was triggered when the back of the participant took off from the chair and the timer stopped when the participant got back to the initial sitting position. Familiarization tests in VR were offered to people to ensure a good understanding of the basic task. An experimenter was always present near the participant during the performance of the task to assure safety.

Two conditions were performed in a randomized controlled trial to avoid any learning effect

• Three trials in reality (Real), in an empty room.
• Three trials in virtual reality (VR), in an environment representing a stationary train.

During the realization of the whole task, two basic variables of locomotion were measured using a camera placed in the corner of the room with a manual post-experimentation video analysis (filmed with a GoPro 1920 p × 1080 p, 60 fps; time was displayed on the video player MPC-HC); the variable “time” representing the main performance evaluation indicator and the variable “number of steps” corresponding to the gait adjustments, i.e., the locomotion strategy. Each step was considered as a heel contact, even if trampling, and this was also applicable for the turnaround phases, during which postural adjustments and weight transfers were necessary and therefore counted as steps. There are several primary domains of spatiotemporal gait performance identified in the literature but considering the placement constraint of the camera used, only the number of steps and the time taken for performing the whole task have been measured accurately and reliably.

### Set up of the immersive virtual environment

**FIGURE 1:**
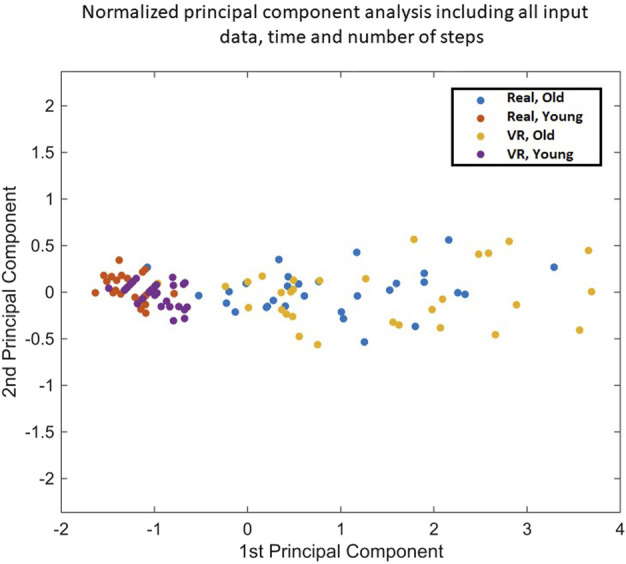
| Normalized principal component analysis, including all input data, time, and number of steps.

The virtual application was developed on the Unity game engine. The virtual reality device was based on the use of an HTC Vive HMD (Framerate: 90 Hz, 2160 × 1200 combined pixels, 110° field of view, 470 g) and a computer with a “Nvidia Geforce GTX 1070 GPU” to allow IVR software to run smoothly. Participants were immersed in a virtual environment (VE) representing a stationary wagon-bar train (Figure 1). This scenario was relevant to the task requested, with a virtual train chair located at the same position as the real chair, and the go back and forth was carried out in the corridor of the train. Participants were asked to perform the turnaround in front of a suitcase placed 3 m away; they were not allowed to go around the suitcase. To facilitate visual information gathering about the location of the Turn-Around, we used a suitcase. This helped compensate for the reduction in vertical field of vision. In the real condition, we placed a 3-m line marker on the ground where the turnaround point was perfectly adjusted to have the same distance as in the VR condition. Finally, participants saw the VE in first-person view and moved into it with visual feedback consistent with the gaze orientation and movements performed. The exposure time was short, greatly limiting the possibility of feeling motion sickness. They had no representation of their body with an avatar, and therefore, had no exproprioceptive visual feedback from their body segments.

### Definition of a functional mobility score

**FIGURE 2:**
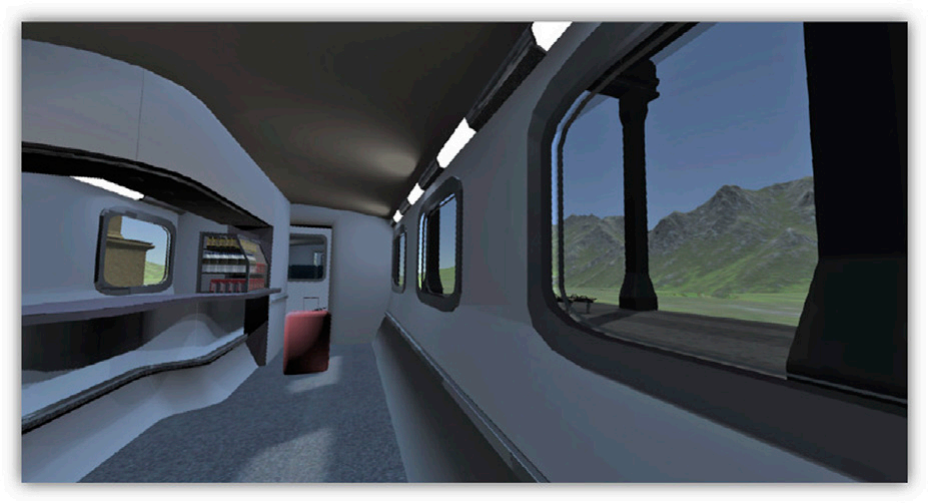
Virtual environment design of a wagon-bar train.

Functional mobility is a way to assess the global health of an individual through their ability to move freely. As defined, functional mobility is the ability to perform basic activities of daily living, such as performing the locomotor task asked of participants, consisting of performing a go back and forth of 3 m, starting and finishing in a sitting position. In this study, a functional mobility score was therefore defined to quantify the ease and efficiency in performing the locomotor task. This score was calculated by applying a normalized principal component analysis (PCA) (Figure 2), combining the two input variables, which were time and number of steps of all participants (the main indicators of locomotion that are highly correlated), in a single explanatory output variable, a functional mobility score.

Explained first Principal Component: eigenvalue = 1.945.

Explained second Principal Component: eigenvalue = 0.055.

Mathematical equation: y=0.9773 * x1+0.227 * x2

This PCA identified a new main indicator explaining 97.73% of the variance, an extremely high score, called the functional mobility score. Indeed, according to the Kaiser criterion, which specifies that only variables with an eigenvalue superior or equal to 1 are retained, only the first component was studied as the eigenvalue was 1.945 The second component was not considered as the eigenvalue was 0.055. Therefore, PCA was used to reduce the dimensionality of the explanatory variables.

### Statistical analysis

The “number of steps” and “time” raw data were preprocessed by averaging the three trials for each participant in each condition. All statistical processing steps used the average values of the three trials calculated and MATLAB software.

First, including all participants, linear regressions between “number of steps” and “time” for each condition were calculated and compared by an analysis of covariance (ANCOVA). A PCA was applied to determine the new variable, called the functional mobility score. As the values between young people and older people were heterogeneous and distinct, a variation of the mobility score in VR was calculated and expressed the evolution of the mobility score in VR compared with reality as a percentage. This allowed young and older people to be compared accurately.

Normality was tested using the Lilliefors test. The distribution of the data followed a normal law for the mobility score. Two-by-two interindividual comparisons were made using Student’s t-test for the functional mobility score, with a significance threshold set at 0.05.

## Results

**FIGURE 3:**
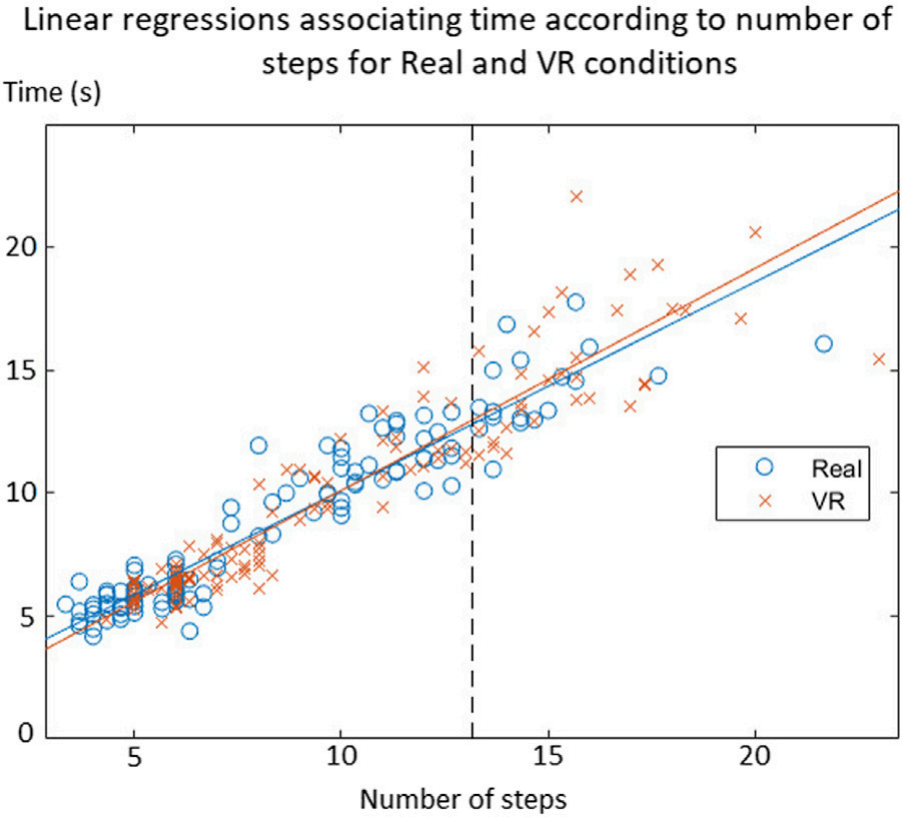
Linear regressions associating time according to the number of steps for Real and VR conditions.

Both linear regressions calculated expressed time according to the number of steps in the “Real” and “VR” conditions (Figure 3). The correlation coefficient in Real was *r* ^2^ = 0.914; the correlation coefficient in VR was *r* ^2^ = 0.913. Correlation coefficients were high for both conditions. ANCOVA showed no significant differences between these two linear regressions (*p* = 0.77). The ANCOVA model used was as follows: y = −3,0208 + 0,87525. x + *ε*

This means that the relationship between time and the number of steps was equivalent in Real and VR for all participants. Considering that both linear regressions were equivalent between “Real” and “VR” conditions, it was justified to group the data to retain a single regression model, which has been used for the determination of the functional mobility score by applying a PCA.

**FIGURE 4:**
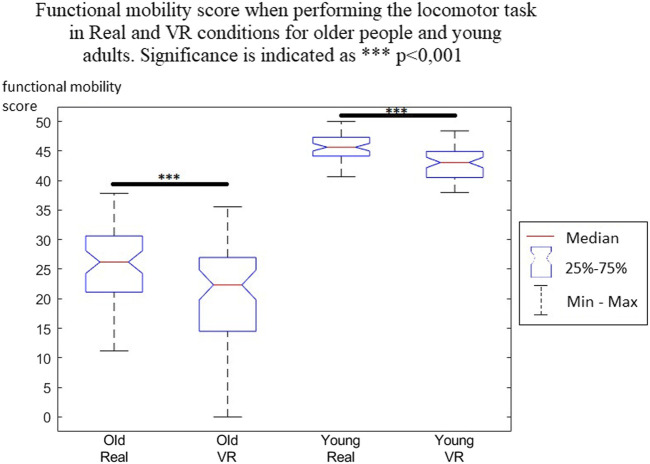
Functional mobility score when performing the locomotor task in Real and VR conditions for older people and young adults. Significance is indicated as ***p < 0.001.

Statistical results showed a significative decrease in the functional mobility score for older people (*p* < 0.001) and young adults (*p* < 0.001) in the VR condition compared with the Real condition (Figure 4). This means that there was a performance decrease in the VR condition for both populations, confirming an IVR effect on motricity.

**FIGURE 5:**
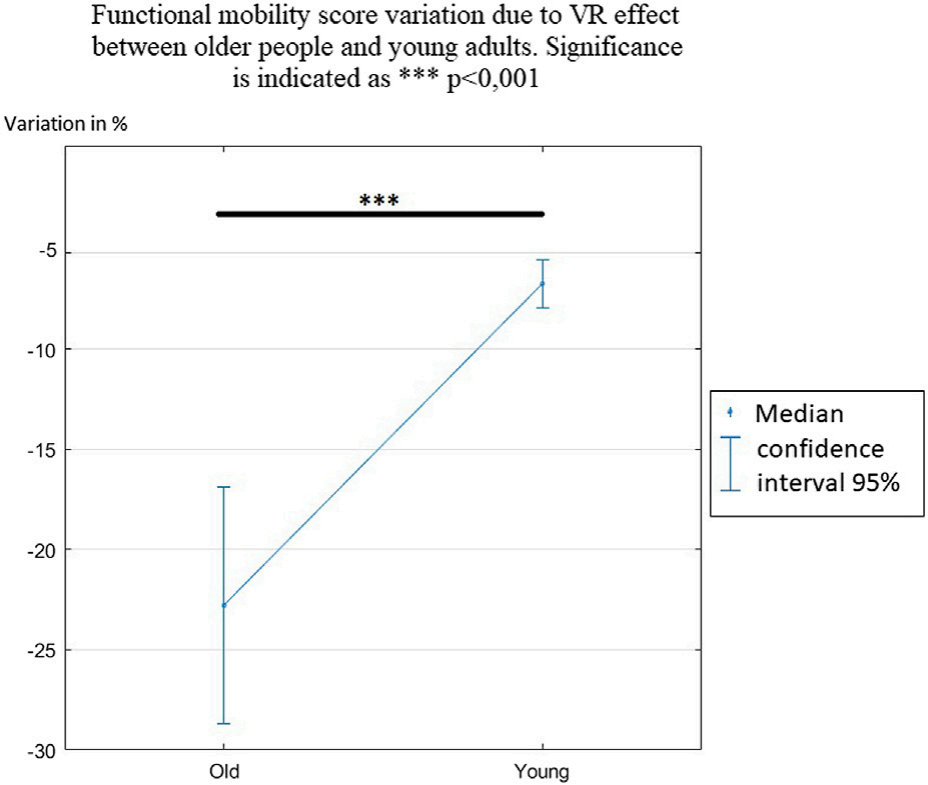
Functional mobility score variation due to the VR effect between older people and young adults. Significance is indicated as ***p < 0.001.

The variation of the functional mobility score reflected the percentage difference in the functional mobility score in VR compared with that in the Real condition (Figure 5). A negative variation showed a decrease of this score, meaning a decrease in locomotor performance. In older people, the median decrease was 22.79%, while in young people it was 6.75%.

Student’s t-test, which compared the variation in the functional mobility score between older people and young adults, showed a significant difference (*p* < 0.001). This means that the VR effect on the functional mobility score was higher for older people, who reduced their motor performance more significantly when performing the locomotor task. Another interesting result was that the intervariability of the score was significantly higher in older people than in young adults.

## Discussion

The objective of this study was to determine the influence of an immersive virtual scenario on functional mobility according to age; this technology could allow for contextualized, standardized, and controlled situations that could serve as an immersion support during a motor evaluation. The main results showed that the locomotion pattern seemed to be preserved in virtual reality but with a significative decrease in the functional mobility score for both older people and young adults. However, this decrease in locomotor performance was more pronounced in older people; therefore, the VR effect was accentuated by the aging process.

This study confirmed the existence of an effect of immersive virtual reality on the motor skills of older people and young adults, more specifically on a functional mobility score that was defined as the combination of the variables time and number of steps when performing the locomotor task. This score decreased significantly in virtual reality for both populations, reflecting a change in the motor strategies used. These results are consistent with several studies that had already shown that performing the same task in virtual reality compared with reality modified motor performance (Fink, Foo and Warren, 2007; Menegoni et al*.*, 2009; Morel et al*.*, 2015; Agethen et al*.*, 2018; Almajid et al*.*, 2020; Muhla et al*.*, 2021). Even though participants need a longer time to complete the same task in virtual reality, they are able to finish it without stopping or falling; this locomotor behavior can be explained by the visuomotor behavior being essentially similar between reality and virtual reality (Gérin-Lajoie et al*.*, 2008; Bühler and Lamontagne, 2019), which confirms that walking in a virtual environment is performed automatically and similarly to walking in a real environment (Fuchs, 2018). Here, there was a correlation between the number of steps and time, which is statistically the same in virtual reality and reality for the whole population (Figure 3). This means programming and execution centers of locomotion seemed to function in the same way for moving in virtual reality, whether the participant is young or old. It implies that the participant analyzed the virtual environmental context and made behavioral decisions following the same process as an analysis of a real environment. On the other hand, it suggest that general motor patterns are preserved but parameterized differently, resulting in an adaptation of the motor strategies used, such as the reduction of pace, for example,. This result is in accordance with a study showing that the same gait adaptability behavior is used between real and virtual environments (Delbes et al*.*, 2022). The use of this technology is thus relevant in the assessment of mobility or motor capacities. However, it is necessary to better understand the effects of immersive virtual reality on the motor strategies used according to age.

The main aim of this study was to compare the age-related evolution of a functional mobility score to determine the locomotor performance in an immersive virtual reality environment compared with a real environment. The characteristics of motor control, balance, and walking affecting motor skills are partly age dependent, linked to the consequences of aging on functional capacities. The descriptive data clearly showed these differences in motor behavior when performing the locomotor task in real conditions; the older people having a functional mobility score approximately half that of young adults. Older people compensate for their reduced physical abilities by adopting a safer and more cautious motor strategy that reduces the energy cost of their movements (Barak, Wagenaar and Holt, 2006).

The effects of immersive virtual reality on locomotor performance existed for both populations, as described previously, and generated a decrease in the functional mobility score. These effects are related to the characteristics of the virtual experience and the technological limitations that prevent the simulation of a fully ecological environment, particularly the weight of the HMD on the head, which imposes perceptual and biomechanical constraints (Patterson et al., 2006), and the graphics quality, which is still largely improvable. Additionally, the complete isolation from the physical world and the lack of stimulated multimodal sensory inputs lead to a decrease in motor performance (Muhla et al., 2021; Renaux et al., 2022). The results of this study nevertheless showed that locomotor performance decreases were significantly accentuated by aging, confirming our hypothesis. Indeed, the evolution of the functional mobility score in virtual reality was significantly more reduced in older people (22.79%) than in young adults (6.75%). However, this global result must be slightly qualified within the older population due to important interindividual differences. In young adults, on the other hand, there was a similar evolution of the functional mobility score in virtual reality, with a small interquartile range.

A few explanatory hypotheses can be put forward to explain why older people reduced their locomotor performance significantly more in virtual reality. First, living an immersive virtual reality experience using an HMD leads to a reduction in visual information from the virtual environment, especially in terms of peripheral vision as the HMD has a reduced field of view compared with the usual binocular field of view. Numerous studies have shown that a reduced field of view influences mobility, resulting in a decrease in walking speed. In particular, the lower peripheral field of vision and central vision are mainly used by older people (Turano et al., 2004). Indeed, once a path is determined, visual information from the central and inferior peripheral visual fields allows a continuous update of the spatial environment (Marigold, 2008). Gibson (1954) proposed that patterns in the optical network defined by the visual field are stimuli for locomotion control because they provide information about the direction and speed of self-motion (Gibson, 1954). Vision provides the only direct measure of motion that is useful for regulating locomotion speed and direction (Warren, 1995). However, several sources of sensory information are integrated for locomotion and balance in order to adapt motor behavior in an environment as optimally as possible, but older people perform this continuous sensory weighting less efficiently than younger adults (Horak, Shupert and Mirka, 1989). The decrease in visual information is particularly detrimental for older people because advanced age is linked to a greater visual dependence on sensory integration to control postural balance (Agathos et al*.*, 2017). This means that older people must have had more difficulty in the virtual environment, which reduced the visual information available, and thus they had to rely on safe compensatory strategies to avoid failing the task, such as reducing step length and the rate.

On the other hand, always in connection with the reduction in visual information and the continuity of the aforementioned arguments, it is commonly admitted that older people look more at the ground when they move than young adults (Anderson et al*.*, 1998), particularly to get feedback on their foot placement. This is exproprioceptive visual information about limb position and movement, which is used on an ongoing basis to refine the limb swing trajectory (Patla, 1997). The visual exproprioceptive information refers to the information of the body in relation to the environment (Gibson and Carmichael, 1966) and may be the critical piece of sensory information that explains why improvements in walking can be achieved with visual step cues. This has been highlighted in patients with Parkinson’s disease, for example, (Vitório et al*.*, 2014). This information is distinguished from visual exteroception, which refers specifically to information about the environment (Lee, 1978). Visual exproprioceptive information about the position of the lower limbs in relation to the environment can be associated with proprioceptive feedback from the lower limbs and with an efference copy of the motor command. This information is then used to correct the posture. In the virtual experiment carried out in this study, participants did not have visual exproproprioceptive feedback from their own body segments and, in particular, from their own steps because there was no body representation through the use of an avatar. However, older people essentially need vision at a particular time in the step cycle to effectively plan future step movements, while younger adults are much less affected by this disability (Chapman and Hollands, 2006), particularly because they can rely on proprioceptive information to compensate, whereas older people find this more difficult to use. Thus, the inability to see their own body segments, which are normally used to continuously refine their locomotion and posture, probably played an important role in the greater reduction in the functional mobility score. Studies have shown that the embodiment over an avatar colocated with the body segments significantly improves the precision of movements in the environment (Pan and Steed, 2019). The lack of an avatar, and thus of exproprioceptive visual feedback, seems to have affected more the older people who rely mainly on vision to place their body segments. For example, during locomotion, the minimum foot clearance, defined as the minimum distance between the ground and the foot in the middle of the swing, increased significantly when ongoing exproprioceptive visual signals were not available (Graci, Elliott and Buckley, 2009). Thus, the visual exproprioceptive information seemed to be important because individuals modified their motor strategy without it.

It could also be assumed, although it was not measured in this study, that there was a psychological influence of apprehension in using this technology for the first time, which led older people to adopt a more cautious strategy in this new and unknown environment, related to the fear of falling. It would be interesting to measure this in future studies.

In conclusion, there is an age-related effect of immersive virtual reality on the decrease in locomotor performance. Indeed, this study showed that older people were significantly more affected by a decrease in their functional mobility score than young adults in the virtual environment. As older people are more visuo-dependent, the results are most likely explained by the reduction in visual information available in the virtual environment, linked with the reduced field of view in the HMD and the lack of exproprioceptive visual information in real time about the placement of body segments. Therefore, an avatar should be integrated in future studies to partially compensate for this lack of information.

## Data Availability

The original contributions presented in the study are included in the article/Supplementary Material, further inquiries can be directed to the corresponding author.
